# Analysis of survival and hatching transcriptomes from potato cyst nematodes, *Globodera rostochiensis* and *G*. *pallida*

**DOI:** 10.1038/s41598-017-03871-x

**Published:** 2017-06-20

**Authors:** Marc-Olivier Duceppe, Joël Lafond-Lapalme, Juan Emilio Palomares-Rius, Michaël Sabeh, Vivian Blok, Peter Moffett, Benjamin Mimee

**Affiliations:** 1 Agriculture and Agri-Food Canada, 430, Boulevard Gouin Saint-Jean-sur-Richelieu (Québec), J3B 3E6 Québec, Canada; 2 Département de Biologie, Université de Sherbrooke, Sherbrooke, J1K 2R1 Canada; 3 Cell and Molecular Sciences, The James Hutton Institute, Invergowrie, Dundee, DD2 5DA United Kingdom; 4 Canadian Food Inspection Agency, Ottawa Laboratory Fallowfield (OLF), 3851 Fallowfield Road, Ottawa, Ontario K2H 8P9 Canada; 5 Institute for Sustainable Agriculture (IAS), Spanish National Research Council (CSIC), Avenida Menéndez Pidal s/n, 14004 Córdoba, Campus de Excelencia Internacional Agroalimentario ceiA3 Spain

## Abstract

Potato cyst nematodes (PCNs), *Globodera rostochiensis* and *G*. *pallida*, cause important economic losses. They are hard to manage because of their ability to remain dormant in soil for many years. Although general knowledge about these plant parasitic nematodes has considerably increased over the past decades, very little is known about molecular events involved in cyst dormancy and hatching, two key steps of their development. Here, we have studied the progression of PCN transcriptomes from dry cysts to hatched juveniles using RNA-Seq. We found that several cell detoxification-related genes were highly active in the dry cysts. Many genes linked to an increase of calcium and water uptake were up-regulated during transition from dormancy to hydration. Exposure of hydrated cysts to host plant root exudates resulted in different transcriptional response between species. After 48 h of exposure, *G*. *pallida* cysts showed no significant modulation of gene expression while *G*. *rostochiensis* had 278 differentially expressed genes. The first *G*. *rostochiensis* significantly up-regulated gene was observed after 8 h and was coding for a transmembrane metalloprotease. This enzyme is able to activate/inactivate peptide hormones and could be involved in a cascade of events leading to hatching. Several known effector genes were also up-regulated during hatching.

## Introduction

Potato cyst nematodes (PCNs), *Globodera rostochiensis* and *G*. *pallida*, are major plant-parasitic nematodes of potato and are found infesting fields alone or as mixtures of both species^1^. They are present in the major world potato production areas and are quarantine organisms in many countries^2, 3^. Yield losses are usually proportional to initial soil contamination^4, 5^ and are estimated at 2 t/ha of potatoes for every 20 eggs/g of soil^6^. For *G*. *rostochiensis*, an initial population density as low as 0.1 J2/cm^3^ of soil can significantly reduce potato yields^7^. These damages will depend on several factors including soil type, interactions with microorganisms, differences in husbandry, cultivars, weather and potential yields between sites^8^. However, yield losses over 50% have been reported^8^. PCNs can also attack other crops (tomato, eggplant) and several Solanaceaeous weeds such as nightshades, which can serve as reservoirs^9, 10^. *G*. *rostochiensis* and *G*. *pallida* are members of the *Heteroderidae* and originate from South America. They were probably introduced to Europe along with potato breeding material around 1850^11^.

Like other specialized parasites, the PCN life cycle is synchronized with their hosts to optimize the chances of successful invasion^12^. This synchrony is possible because PCN unhatched juveniles have the ability to remain dormant until a stimulus from a host is perceived, indicating favourable conditions for hatching. PCN eggs are trapped inside the dead female body, forming the cyst structure, and can survive in soil for over 20 years^13^. Hatching occurs in response to root diffusate from a suitable Solanaceae growing nearby. However, some eggs will only hatch on restimulation, a strategy to increase population persistence throughout growing seasons and to lower competition between hatched juveniles^12^. Variable spontaneous hatching also occurs, depending on field conditions^14^.

The PCN hatching process consists of three main steps: increase of the eggshell permeability, activation of the larva and eclosion^15^. Trehalose inside the eggs is associated with survival and hatching. The osmotic stress caused by the accumulation of this sugar will inhibit locomotion and induced quiescence, thus providing protection against environmental stresses^16^. The hatching process starts with a permeability change of the eggshell lipid layer involving Ca^2+^ ^17^, and subsequent leakage of trehalose in response to host root diffusates^18^. With the loss of osmotic pressure, water uptake will allow juvenile larvae to rehydrate and to restore motility. Active larvae will cut the eggshell and hatch. Changes in the lipid content and fatty acid composition of the larvae also occur in the egg after exposure to potato root diffusate^19^. A number of external environmental factors, including host plant root diffusates, soil temperature and moisture, soil oxygen, soil microorganisms, minerals and organic substances, can serve as hatch inducers or can influence hatching^20^. Natural compounds (i.e. solanoeclepin A) and synthetic analogues^21^ and other chemicals such as picrolonic acid, sodium thiocyanate, alpha-solanine, and alpha-chaconine partially stimulate the hatching process, with greater hatching levels for *G*. *rostochiensis* than for *G*. *pallida* ^22^. Using potato root diffusate (PRD), Perry and Beane^23^ showed that a single 5-min exposure was enough to induce hatching of *G*. *rostochiensis* eggs. In contrast, *G*. *pallida* eggs required weekly 5-min exposures to PRD to induce hatching^24^. In an experiment to establish the relationship between soil temperature and PCN hatching, Kaczmarek *et al*.^25^ have shown that *G*. *rostochiensis* hatched more quickly than *G*. *pallida* and that hatching of both species increases with temperature with a peak around 20 °C.

The series of physiological and behavioral events associated with hatching strongly suggest that changes in gene expression may be involved in the process. However, very little is known about which genes are important to cyst survival and hatching. Jones, *et al*.^26^, using differential display as analytical technique, did not find any changes in gene expression linked to exposure to PRD in *G*. *rostochiensis*. On the other hand, they found a few differentially expressed genes (DEGs) associated with cyst survival, but none of them showed significant homology to known sequences. Similarly, Qin, *et al*.^27^ highlighted a few coding sequences by cDNA-AFLP related to *G*. *rostochiensis* cyst survival and pathogenicity.

Other studies have showed indirect observations of increased transcriptional activity during hatching of PCNs. Perry^28^, as well as Atkinson, *et al*.^16^, found an accumulation of secretory granules and an increase of the nucleolus size of the dorsal oesophageal glands of *G*. *rostochiensis* within a few hours of exposure to PRD. Likewise, Blair^29^ found an increase in staining of a nucleic acid specific dye in unhatched second stage juveniles of *G*. *rostochiensis* after three days of exposure to tomato root diffusate (TRD).

A recent transcriptome analysis of *G*. *pallida* has shown that 526 genes were up-regulated at the transition from encysted eggs (containing dormant J2s) and hatched J2 nematodes^30^. This large-scale activation of transcription is indicative of the numerous metabolic changes that are associated with the up-regulation of genes involved in root penetration and the production of other secreted proteins interacting with plant defense mechanisms. Additionally, Palomares-Rius, *et al*.^31^ showed significant changes in gene expression after hydration of quiescent eggs of *G*. *pallida* and after exposure to TRD using a microarray platform. However, this study was done after soaking cysts for 4 days in TRD.

Here, we studied the changes in transcriptomic activity of PCN from dormant to hatched juveniles using RNA-Seq, a high throughput/hi-resolution technique. Two independent studies, one for *G*. *rostochiensis* and one for *G*. *pallida*, were combined to present shared pathways. These studies were designed to capture early gene activation during hatching. The objectives of this work were to analyze the genes involved in hatching, to identify those necessary for survival and to compare gene expression in two closely related species, *G*. *rostochiensis* and *G*. *pallida* during these key events of their life cycle. In a context of pesticide withdrawal, this knowledge is of vital importance for the design of new integrated pest management strategies.

## Results

### *De novo* transcriptome assemblies

RNA-Seq library sequencing yielded 511 M reads for *G*. *rostochiensis* and 213 M reads for *G*. *pallida*. A total of 239 k components were *de novo*-assembled by Trinity from the *G*. *rostochiensis* reads (assembly statistics are summarized in Table S1). This high number was attributable to the presence of sequences from contaminants. In order to remove the contaminating components from the *G*. *rostochiensis* transcriptome, we developed a decontamination method called Contaminant Contigs Removal by Counts (CCRbC, summarized in Material and Methods). This step removed 61% of the components, leaving 93,089 final contigs, about three times more contigs compared to the *G*. *pallida* transcriptome.

In comparison, the *G*. *rostochiensis* reference transcriptome has 14,309 contigs^32^ and the *G*. *pallida* reference transcriptome has 16,417 contigs^30^. Only 19.1% of the *G*. *rostochiensis de novo* contigs had BLAST hits (e-value < 1e^−10^) on its reference transcriptome, but these contigs actually covered 96.9% of the reference transcriptome. For *G*. *pallida*, 70% of the *de novo* contigs had a BLAST hit on its reference transcriptome and these contigs covered 82.7% of the reference transcriptome.

### Differentially expressed genes analyses

**Figure 1:**
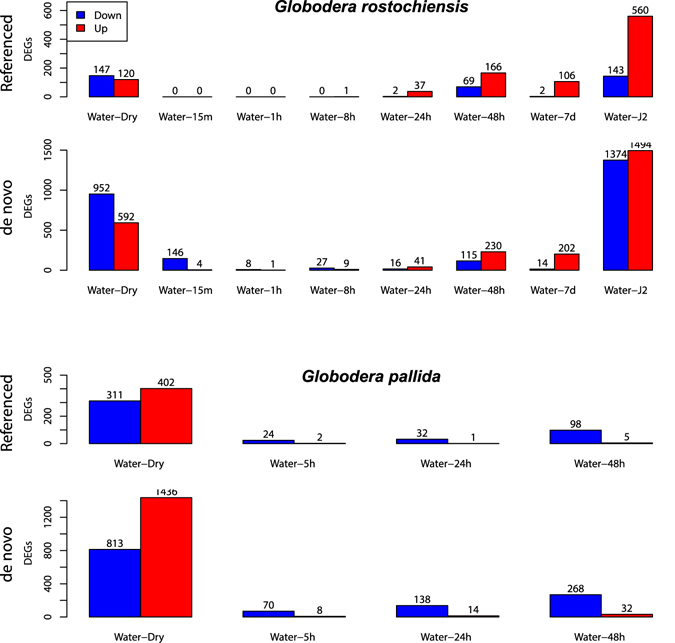
Pairwise counts of differentially expressed genes (DEGs) in each treatment of each transcriptome. The control treatment “water” represents hydrated cyst and time represent the soaking time in PRD/TRD after hydration.

DEGs for *G*. *rostochiensis* and *G*. *pallida de novo* transcriptomes and reference transcriptomes are summarized in Fig. 1 and detailed in Tables S2 to S5. For both PCNs, there were more DEGs found using the *de novo* transcriptomes than the reference transcriptomes. The ratio of up- and down-regulated genes in each treatment was similar between Trinity and reference analyses for both species. However, *G*. *pallida* had more up-regulated than down-regulated genes in the dry cysts and the opposite at all hatching time-points (5 h, 24 h and 48 h of exposure to TRD). Conversely, *G*. *rostochiensis* had more down-regulated than up-regulated genes in the dry cysts and the opposite during hatching (24 h, 48 h and 7 days of exposure to PRD).

### Survival

Many contigs were differentially expressed in the dry cysts in comparison to hydrated cysts. Using the *de novo* transcriptomes to measure gene expression, we found 592 up- and 952 down-regulated contigs for *G*. *rostochiensis* and 1436 up- and 813 down-regulated contigs for *G*. *pallida* in dry cysts (Fig. 1). Interestingly, seven putative survival-related DEGs with similar upregulated expression patterns had homologs in all four transcriptomes (Table 1). Similarly, eight of the genes upregulated after cyst hydration were common to all transcriptomes (Table 2). BLAST results for all these contigs can be found in Supplemental Tables S2–S5.

**Table 1:** Differentially expressed genes (DEGs, P < 0.05, FDR < 0.1) up-regulated in dry cysts common to both *G*. *rostochiensis* and *G*. *pallida* in both *de novo* and reference transcriptomes.

Transcript name for:		
Trinity G. *rostochiensis*		
Reference *G*. *rostochiensis*		
Trinity *G*. *pallida*		
Reference *G*. *pallida*	BLAST results	DEG fold change
comp209610_c0	dihydrodiol dehydrogenase	4.29
G14.T1		4.59
comp35682_c0_seq2		1.52
GPLIN_000780500		1.87
comp238116_c0	thiazole biosynthetic enzyme	3.03
G9808.T1		3.73
comp29709_c0_seq5		1.87
GPLIN_000109200		2.14
comp248107_c0	protein ttr	3.25
G6983.T1		3.73
comp35561_c0_seq4		1.74
GPLIN_000178900		1.74
comp217753_c0	dorsal gland cell-specific expression protein	1.62
G1821.T1		3.03
comp36097_c0_seq6		1.74
GPLIN_000717000		2.83
comp233714_c0	adipocyte plasma membrane-associated protein	2.00
G1457.T1		3.03
comp31390_c0_seq10		1.52
GPLIN_001294200		1.74
comp252050_c0	superoxide dismutase	2.3
G3110.T1		3.25
comp30335_c0_seq3		1.74
GPLIN_000288300		1.87
comp212223_c0	an1-type zinc finger protein	2.14
G9913.T1		2.64
comp28302_c0_seq5		1.41
GPLIN_000417800		1.74

**Table 2:** Differentially expressed genes (DEGs, P < 0.05, FDR < 0.1) up-regulated in hydrated cysts common to both *G*. *rostochiensis* and *G*. *pallida* in both *de novo* and reference transcriptomes.

Transcript name for:		
Trinity *G*. *rostochiensis*		
Reference *G*. *rostochiensis*		
Trinity *G*. *pallida*		
Reference *G*. *pallida*	BLAST results	DEG fold change
comp252939_c1	protein del- isoform a	5.28
G11187.T1		3.25
comp32699_c0_seq2		2.30
GPLIN_000940400		1.87
comp258240_c1	transmembrane cell adhesion receptor mua-3	6.50
G2584.T1		3.25
comp37877_c0_seq2		2.64
GPLIN_000889800		2.00
comp89713_c0	lipase family protein	4.92
G9703.T1		3.25
comp26677_c0_seq1		2.46
GPLIN_000757300		1.74
comp34138_c0	beta-endoglucanase	32.0
G9434.T1		17.15
comp32250_c0_seq8		3.035
GPLIN_000552400		2.00
comp233971_c0	transport and golgi organization-like protein	8.00
G4478.T1		4.59
comp31082_c0_seq4		3.03
GPLIN_000347500		3.03
comp199786_c0	protein unc- isoform b	11.31
G494.T1		4.92
comp36789_c0_seq1		1.87
GPLIN_000299900		1.41
comp257544_c2	four domain-type voltage-gated ion channel alpha-1 subunit	11.31
G4366.T1		5.66
comp37850_c0_seq16		2.00
GPLIN_000712300		2.30
comp242611_c0	protein gcy-9	18.38
G11633.T1		8.00
comp34862_c0_seq2		6.50
GPLIN_000596200		6.96

### Hatching

**Figure 2:**
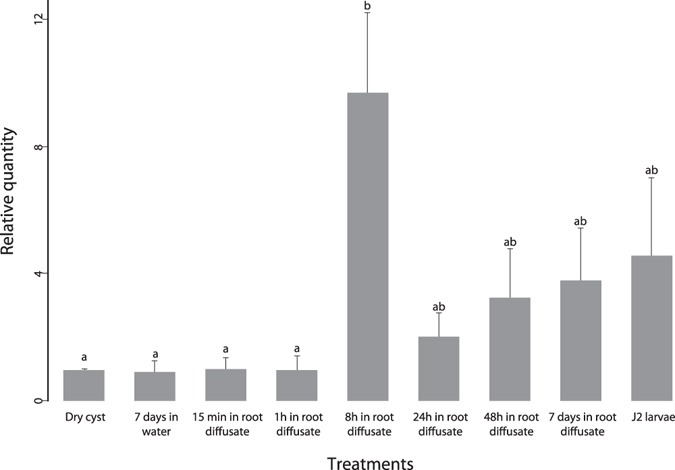
Expression of neprilysin gene *nep-1* by RT-qPCR in dry cysts, hydrated eggs, and hydrated eggs exposed to potato root diffusate (PRD) for 15 min, 1 h, 8 h, 24 h, 48 h and 7 days and hatched J2. Hydrated cyst expression level was use as calibrator. Error bars represent standard error of the mean and significant differences among treatments are indicated by different letters (Tukey).

The first *G*. *rostochiensis* transcript to be significantly up-regulated in both Trinity and reference transcriptomes, i.e. after 8 h exposure to PRD, was encoding for a protein similar to the neprilysin NEP-1 (comp140896_c0, Fig. 1 and Tables S2 and S3). The expression pattern of this gene was confirmed by RT-qPCR (Fig. 2). Also, 39 contigs common to both *G*. *rostochiensis de novo* and reference transcriptomes were significantly up-regulated during hatching treatments (cysts soaked for 8 h, 24 h and 48 h in PRD; Table 3). No DEGs were shared in both *G*. *pallida* transcriptomes in response to hatching treatments (up to 48 h). No hatched J2 were observed across time-course except a few individuals of *G*. *rostochiensis* after 7 days of exposure to PRD.

**Table 3:** Differentially expressed genes (DEGs, P < 0.05, FDR < 0.1) up-regulated after hatching 8 h, 24 h or 48 h exposure to potato root diffusate common to *G*. *rostochiensis de novo* and reference transcriptomes.

Contig *de novo* Contig reference	Blast results	DEG fold change	Up-regulated treatments
comp140896_c0	Protein nep-1	6.7	8 h
G11130.T1		3.6, 3.0, 3.2	8 h, 24 h, 48 h
comp79822_c0	pectate lyase 2	8.5, 11.6	24 h, 48 h
G3826.T1		9.6, 6.4	24 h, 48 h
comp223900_c0	fatty acid elongation protein 3	7.6, 9.1	24 h, 48 h
G9188.T1		6.1, 3.0	24 h, 48 h
comp233971_c0	transport and golgi organization-like	7.6, 7.3	24 h, 48 h
G4478.T1		5.7, 3.2	24 h, 48 h
comp197008_c0	extracellular solute-binding protein family 1	7.0, 6.7	24 h, 48 h
G5298.T1		5.5, 3.5	24 h, 48 h
comp239365_c0	cre-mig-17 protein	6.6, 7.6	24 h, 48 h
G6254.T1		4.4	48 h
comp250236_c1	acid phosphatase-1	5.7, 9.4	24 h, 48 h
G3528.T1		8.1, 2.5	24 h, 48 h
comp252640_c1	arabinogalactan endo- -beta-galactosidase	5.7, 8.1	24 h, 48 h
G7269.T1		6.4, 3.5	24 h, 48 h
comp241201_c2	histidine acid phosphatase family	5.3, 5.6	24 h, 48 h
G8616.T1		4.9, 3.8	24 h, 48 h
comp146670_c0	pectate lyase 1	5.0, 6.8	24 h, 48 h
G7095.T1		6.7, 4.4	24 h, 48 h
comp258474_c0	protein cht-2	4.9, 5.3	24 h, 48 h
G11848.T1		8.1, 4.3	24 h, 48 h
comp253737_c1	sodium bicarbonate transporter-like protein 11	4.7, 6.2	24 h, 48 h
G10850.T1		4.7, 2.8	24 h, 48 h
comp205597_c0	alpha-carbonic anhydrase	4.7, 6.2	24 h, 48 h
G4741.T1		7.0, 3.6	24 h, 48 h
comp249939_c0	expansin partial	4.0, 5.9	24 h, 48 h
G9520.T1		5.9, 3.8	24 h, 48 h
comp82167_c0	phosphoglycerate mutase	3.8, 8.0	24 h, 48 h
G4316.T1		6.5, 7.3	24 h, 48 h
comp242049_c0	glutamine synthetase	3.4, 3.7	24 h, 48 h
G3175.T1		1.9	48 h
comp212021_c0	peptidase c13 family protein	8.0	48 h
G6661.T1		5.6	48 h
comp254346_c0	lysosomal protective	8.0	48 h
G926.T1		3.4	
comp256008_c0	protein mlt-7	7.6	48 h
G307.T1		3.2	48 h
comp257944_c0	tartrate-resistant acid phosphatase type 5-like	6.6	48 h
G13014.T1		6.0	48 h
comp231807_c0	c52 protein	5.9	48 h
G5991.T1		8.2	48 h
comp250073_c0	hypothetical protein Aave_2802	5.5	48 h
G9300.T1		5.6	48 h
comp184777_c0	protein fat- isoform a	5.1	48 h
G5119.T1		3.5	48 h
comp252939_c0	protein del- isoform a	4.5	48 h
G11187.T1		3.1	48 h
comp242752_c0	beta-endoglucanase	4.4	48 h
G7081.T1		6.7, 5.7	24 h, 48 h
comp258555_c1	ghf5 endo-beta-glucanase precursor	4.3	48 h
G6471.T1		4.6	48 h
comp220907_c0	hypothetical protein LOAG_17131	7.8	8 h
G12000.T1		2.2	48 h
comp171900_c0	hydroxyacyl-coenzyme a mitochondrial precursor	4.1	48 h
G7218.T1		2.6	48 h
comp219369_c0	beta- levanase invertase	3.9	48 h
G10382.T1		3.7	48 h
comp234017_c0	transmembrane amino acid transporter	3.9	48 h
G1410.T1		1.7	48 h
comp208748_c0	cathepsin z precursor	3.8	48 h
G8230.T1		3.7, 2.2	24 h, 48 h
comp249497_c3	rbp-1 protein	3.8	48 h
G11341.T1		6.4, 4.4	24 h, 48 h
comp235317_c0	protein ugt-49	3.7	48 h
G7585.T1		2.2	48 h
comp250308_c0	n-acetylated-alpha-linked acidic dipeptidase	3.7	48 h
G6374.T1		3.2	48 h
comp204787_c0	Protein C36E8.1	3.5	48 h
G10574.T1		1.4	48 h
comp248143_c0	protein nep- isoform a	3.2	48 h
G5673.T1		13.1, 7.7	24 h, 48 h

### DEGs clustering

**Figure 3:**
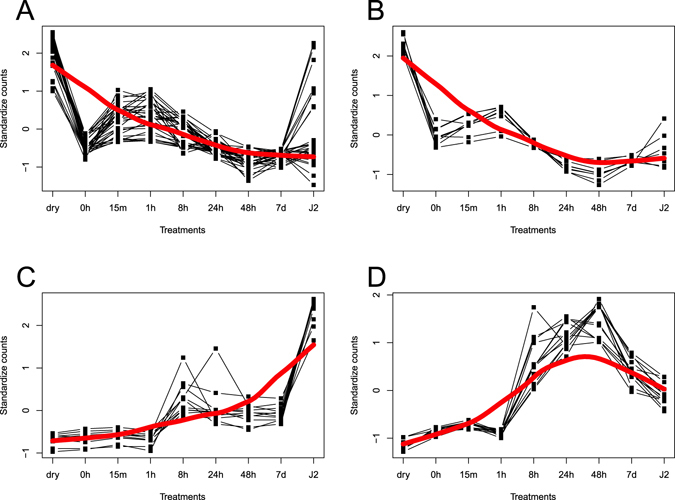
*G*. *rostochiensis* expression clusters of interest harboring differentially expressed genes (DEGs) identified with the Trinity transcriptome. (**A**) Cluster containing the *trehalose 6-phosphate synthase* gene. (**B**) Cluster with a survival-specific expression pattern. (**C**) Cluster containing the *nep-1* gene. (**D**) Cluster with a hatching-specific expression pattern.

The 4,094 unique DEGs obtained with the *G*. *rostochiensis* Trinity transcriptome were clustered in 195 groups. Among them, one cluster showed a clear survival-related expression pattern (Fig. 3B), i.e. high expression levels in dry cysts followed by a decrease in all other treatments. This cluster contained 10 DEGs listed in Table S7. Similarly, one other cluster had a clear hatching-specific expression pattern (Fig. 3D) with low expression levels in dry and hydrated cysts followed by an increase in expression in early contact to PRD, then a plateau and finally a decrease in expression in the hatched J2s. The 13 DEGs from this cluster were up-regulated in at least one hatching treatment (Table S9).

Clusters were also searched for candidate genes involved in survival (*trehalose 6-phosphate synthase*; Fig. 3A) and hatching (*NEP-1*; Fig. 3C). The cluster containing the *trehalose 6-phosphate synthase* had 31 DEGs up-regulated in dry cysts (Table S6). The cluster with the gene encoding for NEP-1 (Fig. 3C), was up-regulated in both *G*. *rostochiensis* transcriptomes in response to hatching factors, and harboured 11 genes (Table S8).

## Discussion

Throughout their coevolution with their hosts, cyst nematodes have developed remarkable abilities to ensure reproduction success and species persistence. One of the most impressive strategies is the ability of potato cyst nematodes, *Globodera rostochiensis* and *G*. *pallida*, to synchronize their hatching with the presence of a suitable host and to survive in soil for several years^13^. Very little is known about the genetic control behind long-term dormancy and hatching. In this work, we have highlighted important genetic pathways that are activated during these key life stages using RNA-Seq. Sequence contamination from soil/cyst microorganisms was found to be a big challenge for data analysis. For *G*. *rostochiensis*, more than 60% of the transcripts obtained were contaminant sequences. A simple yet efficient decontamination algorithm (CCRbC) was developed and applied to remove most of these contaminating sequences without losing important information. Indeed, a horizontal coverage of 96.9% was obtained by aligning the remaining transcripts to the reference transcriptome.

During dormancy, cyst nematodes are anhydrobiotic, surviving almost completely desiccated^33^. In addition, to provide a physical protection, the cyst envelope and eggshell slow down the water loss rate during desiccation, which is thought to be very important for cryptobiosis survival^34^. Trehalose accumulation inside the larvae body is also important for long-term survival. It may replace bound water by attaching to polar side groups of proteins and phospholipids, thus maintaining the balance between hydrophilic and hydrophobic forces acting on the molecules and preventing them from collapsing^15^. In this study, we have found that the expression of a *trehalose 6-phosphate synthase* gene was up-regulated in dry cysts in comparison with hydrated cysts in *G*. *rostochiensis* (Table S8). The gene encoding the enzyme with the opposite biochemical function, *trehalase*, which catalyzes the conversion of trehalose to glucose, was found to be up-regulated in dry cysts of *G*. *pallida*. This observation may be attributable to the fact that after its synthesis in early phases of cryptobiosis, trehalose will later serve as an energy source. Overexpression of a trehalase in *G*. *pallida* could reflect its use. In the specific case of *G*. *pallida*, the cysts were stored at 4 °C, in order to treat for diapause, for more than one year. Thus, they could have started to use their reserves in order to survive longer. This could also be one of the reasons behind the up-regulation pattern in *G*. *pallida* during this stage. Our results reflect the importance of trehalose metabolism during dormancy. Of course, other cellular mechanisms are required to ensure cyst survival during extended desiccation periods^34^.

The desiccation state found in dormant cysts impairs normal reactive oxygen species (ROS) detoxification mechanisms^35^. Molecules such as superoxide $({{\rm{O}}}_{2}^{\cdot -})$, hydroxyl (^**·**^OH) radicals and peroxide (H_2_O_2_) are highly reactive and can damage nucleic acids, proteins and lipids, leading to premature aging or death of cells^36^. Antioxidants are the main molecules capable of balancing ROS levels^35^. Interestingly, we found that several enzymatic antioxidant pathways were up-regulated in dry cysts. One of the most common superoxide radical scavengers, *superoxide dismutase* (comp252050_c0), as well as a *dehydrogenase* (comp209610_c0), were indeed up-regulated in dry cysts (compared to hydrated cysts) in both nematode species, within all four transcriptomes (Table 1). Moreover, a gene coding for a protein similar to the THI4 thiazole biosynthetic enzyme (comp238116_c0, Table 1) was also up-regulated in dry cysts in both nematodes in both *de novo* and reference transcriptomes. Thiazol is the thiamin (vitamin B_1_) precursor. Since vitamin B_1_ has strong antioxidant properties, it could play an important ROS detoxifying role under anhydrobiotic conditions^37^. Most animals do not have the machinery to synthesize complex B vitamins as they can easily find it through their diet. Complex B vitamin biosynthesis genes are found in other cyst nematodes. For example, Craig, *et al*.^38^ found vitamin B_6_ biosynthesis genes in *Heterodera glycines*, the soybean cyst nematode. Additional genes involved in vitamin B_1_, B_5_ and B_7_ biosynthesis with evidence of horizontal gene transfer from bacteria are also found in *H*. *glycines* ^39^. The authors suggested that they could have a protective role for the nematode through their antioxidant properties. These genes were also recently identified in *G*. *pallida* ^30^ and *G*. *rostochiensis* ^32^ genomes.

Another gene (comp204557_c0; Table S7) coding for a selenoprotein (thioredoxin) was overexpressed in *G*. *rostochiensis* dry cysts. This protein also harbours antioxidant properties and was found to play an important role in aging and longevity in different organisms^40–42^. Selenoproteins contain the rare amino acid selenocysteine, encoded by the UGA codon usually coding for translation termination, combined with a special mRNA structure called *selenocysteine insertion sequence* ^43^. Interestingly, thioredoxin reductase is the only selenoprotein ever reported in nematodes^44^ and one of the only enzymes with peroxidase activity known in nematodes. This gene, which showed the highest fold change in our study, has been labeled essential for life in many organisms and is currently a promising target for antiparasitic drugs development against nematodes in humans^45^. Also of special interest, several genes implicated in post-transcriptional regulation and coding for RNA-binding proteins and histone deacetylases were up-regulated in dry cysts.

Both PCNs showed important changes in gene expression in response to hydration. Many of those changes were related to calcium, which is essential for PCN hatching^17^. Our results further support the importance of calcium in early steps of hatching. Among the up-regulated genes (Table 2), *gcy-9* (comp242611_c0) coding for a guanylyl cyclase was of special interest because it is part of a signaling cascade activated by low levels of intracellular calcium leading to the synthesis of cyclic guanosine monophosphate (cGMP), which in turn allows the entry of calcium into the cell^46^. Atkinson, *et al*.^16^ previously showed that intracellular levels of cyclic adenosine monophosphate (cAMP) and cGMP influence hatching of *G*. *rostochiensis*. An increased level of cGMP caused by *gcy-9* overexpression in response to hydration could prepare the cells for a better reactivity to hatching factors, which act in a calcium-mediated way^47^.

Overexpression of the gene encoding the transmembrane protein four domain-type voltage-gated ion channel alpha-1 subunit (comp257544_c2) could also play a role by restoring the permeability of the cell membranes to calcium, as well as the overexpression of the *del* gene coding for a cation channel protein (comp252939_c1). The gene *mua-3* (comp258240_c1) was also up-regulated during hydration, which is predicted to have a calcium ion-binding activity. Palomares-Rius, *et al*.^31^ also found that expression of many transmembrane transporter genes was significantly increased when hydrated *G*. *pallida* cysts were exposed to TRD.

Effector genes were upregulated during the water uptake phase, like a *beta-endoglucanase* (comp242752_c0) known to be an important effector for host root infection^48^. Goellner, *et al*.^49^ also found that expression of this gene was increased prior hatching of *Globodera tabacum* eggs.

In comparison to *G*. *rostochiensis*, *G*. *pallida* eggs normally take longer to hatch in response to root diffusate^25, 50^. Our results are consistent with this observation, with the first few DEGs observed after 48 h of exposure to TRD for *G*. *pallida*. In contrast, by 48 h of exposure to PRD, 278 different DEGs were upregulated in *G*. *rostochiensis*. For *G*. *pallida*, Palomares-Rius, *et al*.^31^ confirmed that several hundred genes were differentially expressed after 4 days of exposure to TRD.

The first DEG was observed after 8 h of exposure to PRD in *G*. *rostochiensis*, a gene coding for a neprilysin (comp140896_c0; Table 3), was up-regulated whether the reference or *de novo* transcriptomes was used to measure gene expression. Neprilysins (NEPs) are transmembrane zinc-metalloproteases that are well conserved throughout the animal kingdom. They were first identified in nematodes by Sajid and Isaac^51^. NEPs are able to hydrolyse peptide bonds at the N terminus of hydrophobic amino acids of a variety of substrates (e.g. enkephalins, tachykinins, neurotensins) thereby not only allowing the degradation of peptides, but also the post-transcriptional modification of inactive precursor peptides^52^. In *Caenorhabditis elegans*, NEP-1 is involved in locomotion and pharyngeal pumping and is highly expressed prior to hatching^52^. More than 20 putative neprilysin genes were identified in *C*. *elegans* ^53^. Here, we found 11 different transcripts for NEPs. Many of them were highly expressed during the early phases of the life cycle (Fig. S1), although stronger expression levels were observed in the later stages. The homolog of this gene in *G*. *pallida* (GPLIN_000276000) is also up-regulated 4 days after TRD exposure^31^, which points to a common hatching mechanisms between both species, but later in *G*. *pallida*. Other Zn^2+^-metalloproteases could also play a significant role in hatching, such as the matrix metalloproteinase in *Heterodera glycines* (Hg-MMP) identified by Kovaleva, *et al*.^54^.

The gene *cht-2* (comp258474_c0; Table 3) coding for a chitinase was up-regulated at 24 h and 48 h following exposure to PRD. Chitinases catabolize the β-1,4-N-acetyl-D-glucosamine polysaccharide chitin, a compound absent from PCN hosts and only found in the eggshell of plant-parasitic nematodes. Similar endochitinases were previously identified in the soybean cyst nematode, *H*. *glycines* ^55^ and in preparasitic southern root-knot nematodes, *Meloidogyne incognita* ^56^.

Several polysaccharide-degrading enzymes genes were up-regulated during hatching (Table 3). Most of them are essential for plant colonization and prepare the nematode for its infective stage. For example, *beta-endoglucanases* (comp242752_c0 and comp258555_c1), *beta-levanase invertase* (comp219369_c0) and *arabinogalactan endo-beta-galactosidase* (comp252640_c1) are cell wall degradation enzymes overexpressed in response to root diffusate. The arabinogalactan endo-beta-galactosidases, which hydrolyse arabinogalactans found in dicot cell walls, may be specific to plant-parasitic nematodes of the cyst nematode group as it is present in *G*. *pallida* and *H*. *schachtii*, but absent from *M*. *incognita* and *M*. *hapla* ^30, 57^.

Many phosphatase genes were also up-regulated in response to root diffusate at various times of exposure, including *histidine acid phosphatase* (comp241201_c2). The product of this gene catalyses the breakdown of phytate (inositol hexakisphosphate), an important phosphorus storage compound in many plants. A recent study has shown that down-regulation of *myo-inositol phosphate synthase* in plants reduces its susceptibility to cyst nematodes^58^.

Multiple genes coding for peptidases (comp239365_c0, comp250308_c0, comp208748_c0 and comp212021_c0) other than NEPs were also up-regulated during hatching of *G*. *rostochiensis*, using both reference and *de novo* transcriptomes. Some of them are found in *G*. *rostochiensis* oral secretions^59^ and are involved in the hatching process in different nematodes, including *G*. *pallida* in later temporal points^60, 61^. It has been proposed that secreted peptidases could play a role in parasitism in plant-parasitic nematodes^62^. This family of enzymes is known to contribute to host specificity, host range and virulence in animal-parasitic nematodes^63^.

Numerous effector genes were up-regulated in response to hatching treatments in both *G*. *rostochiensis* Trinity and reference transcriptomes (Tables 3 and S9), including *expansin* (comp249939_c0), *pectate lyases* (comp146670_c0 and comp79822_c0) and *rbp-1* (comp249497_c3). Pectate lyases are essential for breaking down plant cell walls and were believed to be absent from animals before being found in *G*. *rostochiensis* ^64^. *G*. *rostochiensis* pectate lyases and expansin proteins induce strong phenotypes when expressed *in planta*, suggesting virulence functions^65^. RBP-1 is similar to a gene previously identified in *G*. *pallida* by Blanchard, *et al*.^66^ that contains a SPRY domain and a signal peptide and was strongly suspected to be involved in parasitism. This protein was later identified as the avirulence factor recognised by the potato resistance protein Gpa2^67^. Combined with the high level of polymorphism found among the Ran binding proteins^68^, this finding suggests that this gene family may be under strong selection pressure to evade recognition by the hosts. In the present study, 66 different transcripts with RBP-1 BLAST results were identified, in accord with the previously described high genetic diversity. Jones, *et al*.^69^ suggested that alternative splicing might be involved to create a high potential for adaptation in this gene.

In conclusion, we showed that the quiescent state of PCN is active in terms of gene expression. Many genes involved in cell detoxification are up-regulated in both PCN species. Considerable hatching-associated changes occurred during the hydration phase, based on gene expression evidences, including changes in cell permeability and calcium and cGMP levels. Exposure to root diffusate only affected a small number of genes in the early stages of hatching. Among these genes, several transmembrane metalloproteases genes, including *NEP-1*, were up-regulated and will certainly require further investigation in the future.

## Methods

### Root diffusates

For *G*. *rostochiensis*, potato plants cv. Snowden were grown in perlite, in 2 L containers, until they reached about 15 cm-high. At this point, PRD was harvested once a week, for six consecutive weeks, by the method of Fenwick^70^. Briefly, soil was drenched with tap water until saturation. An extra 50 mL of tap water was then added to the pot and the flowing liquid was collected. The collected liquid was used to repeat this procedure two more times. The final collected liquid was filtered (KenAG, D-547) to obtain PRD. PRD samples were kept at 4 °C in dark plastic bottles until the last one was harvested. Then, all six weekly-sampled PRDs were pooled, freeze-dried and stored at −20 °C. Final volume was recorded prior lyophilization, as well as final weight after lyophilization, for proper PRD reconstitution. PRD was reconstituted from powder with nanopure water at a final concentration of 0.5× and passed through a 0.2 µm filter prior use.

For *G*. *pallida*, tomato plants cv. MoneyMaker were grown in 6-inch pots containing Levington Bio-Multicompost (a mixture of sand, soil and peat). When plants reached 4-weeks old, roots were removed carefully from compost, washed and placed in 250 ml beakers with distilled water. After an incubation period of 4 h, roots were removed and the remaining diffusate filtered using Whatman no. 1 filter paper. Filtered TRD was kept at 4 °C and used within 1 week.

### Sample description

*G*. *rostochiensis* cysts were recovered by flotation^71^ from soil samples collected in the fall 2011 in Saint-Amable (Quebec, Canada). Cysts were stored dried for at least one year in the dark at room temperature prior to hatching experiments. A time course experiment was set up to study the composition of the transcriptome of *G*. *rostochiensis* during quiescence and hatching. The following physiological stages (treatments) were studied: dry cysts, cysts soaked in water for one week (hydration), hydrated cysts soaked in PRD for 15 min, 1 h, 8 h, 24 h, 48 h and 7 d and hatched J2 juveniles. Each cyst sample contained 1000 cysts placed in a mesh bag (Ankom, F57). Cysts were soaked in 30 mL of filtered (0.2 µm) tap water or 0.5× PRD, in a petri dish. Water and PRD were changed every day. All the assays were incubated at 20 °C in an environmental chamber. No hatching occurred during the hydration period. Hatched J2s were harvested daily for a two-week period and pooled for further analysis. Thus, all the treatments, except hatched J2, did not contain any larvae. The experiment was repeated two times.

*G*. *pallida* cysts were from the Lindley population maintained by the James Hutton institute. They were multiplied in glasshouse on the susceptible potato cultivar Désirée growing in a mixture of sand and loam (2:1). Temperature was maintained at 20 ± 1 °C with RH between 60–90%, and a 14-h photoperiod. Cysts were extracted from the soil by flotation in pail by thoroughly mixing infested soil with water. Water was then filtered through a 750 µm-pore sieve nested over a 150 µm-pore sieve. Cysts recovered on the second sieve were kept at 4 °C for at least 3 months. Cysts were soaked in filtered (0.2 µm) tap water for 4 days and subsequently transferred to TRD for 5, 24 or 48 h. All the assays were incubated at 20 °C in an environmental chamber. Cysts were crushed using a tissue homogenizer to release eggs. All the debris were removed from eggs by filtration through a 100 µm-pore sieve nested over a 5 µm-pore sieve. Eggs were concentrated by centrifugation. The experiment was repeated two times.

### Total RNA extraction, library preparation and sequencing

For *G*. *rostochiensis*, cysts soaked in PRD were washed thoroughly with distilled water prior to RNA extraction to remove as much potential contamination as possible. Samples were homogenized in 700 µL of RTL plus buffer with one 6 mm zirconium bead and ~150 µL of 1 mm zirconium beads using the PowerLyzer 24 homogenizer (MO BIO, Carlsbad, CA, USA) and stored at −80 °C until RNA purification. Total RNA was extracted using the RNeasy Plus mini kit (Qiagen, Mississauga, Canada) according to manufacturer’s instructions. Total RNA samples were store at −80 °C prior RNA-Seq library preparation. RNAs were quantified with the NanoDrop 2000 (Thermo Scientific). RNA integrity was assessed with the Bioanlalyzer 2100 (Agilent Technologies) using the RNA 6000 Nano kit. All RNA samples had a RIN value higher than 7 and a 260/230 ratio value over 2.

Library preparation and sequencing were performed at McGill University and Génome Québec Innovation Centre (Montreal, Canada) using the TruSeq RNA sample prep kit v2 (Illumina) and a HiSeq 2000 sequencer (Illumina). For each replicate, all nine samples were multiplexed and sequenced in one lane for 100 bp paired-end reads.

For *G*. *pallida*, total RNA was extracted using RNeasy Plus Micro Kit (Qiagen, Hilden, Germany) following the manufacturer’s instructions. DNA digestion was conducted on column during RNA extraction using RNase-Free DNase set (Qiagen, Hilden, Germany), as recommended. All RNA samples had a RIN value higher than 7 and a 260/230 ratio value over 2. Total RNA was quantified using a 2100 Bioanalyzer (Agilent Technologies) and the Small RNA kit (Agilent Technologies) following the manufacturer’s instructions. Libraries and sequencing were produced and sequenced in Sanger Institute facilities. Illumina transcriptome libraries were produced using polyadenylated mRNA purified from total RNA with size selection using the Caliper LabChip XT.

### Sequence processing, de novo assembly, differential expression analysis and transcript annotation

Reads were trimmed from the 3′ end with a minimal phred score of 30 using the Trimmomatic software^72^ *et al*. Illumina sequencing adapters were removed. Trimmed reads shorter than 32 bp were discarded and orphan reads were kept for the assembly.

The Trinity assembler^73, 74^ was used on normalized trimmed reads (30X coverage) to create *de novo* transcriptomes for both *G*. *rostochiensis* and *G*. *pallida* using default parameters. Minimum contig length was set to 300. A custom script was applied to the Trinity transcriptomes to keep only the longest isoform of each component. Then, we applied an in-house algorithm named Contaminant Contig Removal by Counts (CCRbC) to remove contaminant sequences in the transcriptomes. CCRbC uses as input the counts matrix (n x m) produced by RSEM^75^, where the n contigs are represented by n rows and the r replicates of t treatments are represented by r*t = m columns. The first step is to sum all treatments together for each replicate and for each contig, which resulted in a n by r matrix. Non-contaminant contigs are those that have at least one count for every replicate. Contaminant contigs are removed by cutting rows that contain at least one zero in the n by r matrix. Differential expression (DE; *P* < 0.05 FDR-corrected at 10%) was measured using the DESeq2 Bioconductor package^76^ in R statistical software^77^ using a parametric Wald test. RSEM software was also used to count gene expression on *G*. *pallida* ^30^ and *G*. *rostochiensis* ^32^ reference transcriptomes. Both reference transcriptomes were obtained with the gene prediction software Augustus^78^. DE analysis (DESeq2) was also performed on both reference gene expression matrices produced by RSEM. Contig identification was performed using BLASTx (e-value < 1e-10) against the NCBI nr database. Gene ontology (GO) and InterproScan annotations were done using Blast2GO^79^. BLASTn (P-value < 1e-5) was used to compare the *de novo* transcriptome and the reference transcriptome of both species.

### Clustering

Differently-expressed genes (DEGs) were clustered using the *hclust* function (*cluster* package) and the *cutreeDynamicTree* function (*dynamicTreeCut* package) in R. A matrix containing the fold changes of all DEGs compared in chronological order (e.g. dry-0 h, 0 h-15 m, 15 m-1 h, etc.) was used as clustering input. Expression patterns across treatments, as well as presence of candidate genes, were also used to identify clusters of interest.

### cDNA synthesis for RT-qPCR

Total RNA previously isolated for RNA-seq was treated with DNase I (New England Biolabs). First strand cDNA was synthesized with SuperScript II reverse transcriptase (Invitrogen) from 0.5 μg of total RNA according to the manufacturer’s instruction using oligo (dT)_18_.

qPCRs were performed on a Mx3000 P qPCR system (Agilent Technologies) in a 20 μl reaction volume with 1X TaqMan Universal PCR Master Mix (Applied Biosystems), 1X SYBR green (SYBR Green I Nucleic Acid Gel Stain, Invitrogen), 250 nM of reverse and forward primers and 1 μl of cDNA template. The cycle details were as follow: initial denaturation 95 °C for 10 min, 40 cycles at 95 °C for 20 s and 60 °C for 60 s. A melting curve analysis followed the amplification cycles to examine the specificity of the reaction. Primers are summarized in Table S10. Two biological replicates were used.

The amplification efficiency was calculated using the web-based Real-Time PCR Miner algorithm ver. 4.0^80^. Relative expression analysis of the *nep-1* gene was calculated using the 2^−ΔΔCT^ method^81^. Three genes (GR, PMP-3 and aaRS) reported as stable in all *Globodera* spp. life stages (Sabeh *et al*., in preparation) were used as reference for normalization. Hydrated cysts were used as calibrator treatment to calculate the fold change over the other treatments.

### Data availability

*Globodera rostochiensis* Illumina 100 bp paired-end reads are available through NCBI under the bioproject accession number PRJNA274143. *Globodera pallida* Illumina sequence reads are available through the European Nucleotide Archive (http://www.ebi.ac.uk/ena) under the accession numbers ERR202482-ERR202486 (first repetition) and ERR202488-ERR202492 (second repetition). *G*. *pallida* reference transcriptome is available through the Sanger Institute (ftp.sanger.ac.uk/pub/project/pathogens/Globodera/pallida/).

## Electronic supplementary material

Supplementary Information

Table S2

Table S3

Table S4

Table S5
