# Supplementary material for: Analysis of survival and hatching transcriptomes from potato cyst nematodes, Globodera rostochiensis and G. pallida
Source: Sci Rep. 2017 Jun 20;7:3882. doi: 10.1038/s41598-017-03871-x (PMC5478601; doi:10.1038/s41598-017-03871-x)
Supplement: Supplementary file 1 — Supplementary Information [file 41598_2017_3871_MOESM1_ESM.pdf]

Analysis of survival and hatching transcriptomes from potato cyst nematodes, *Globodera rostochiensis* and *G. pallida*

Marc-Olivier Duceppe, Joël Lafond-Lapalme, Juan Emilio Palomares-Rius, Michaël Sabeh, Vivian Blok, Peter Moffett and Benjamin Mimee

Supplementary information

Figure S1

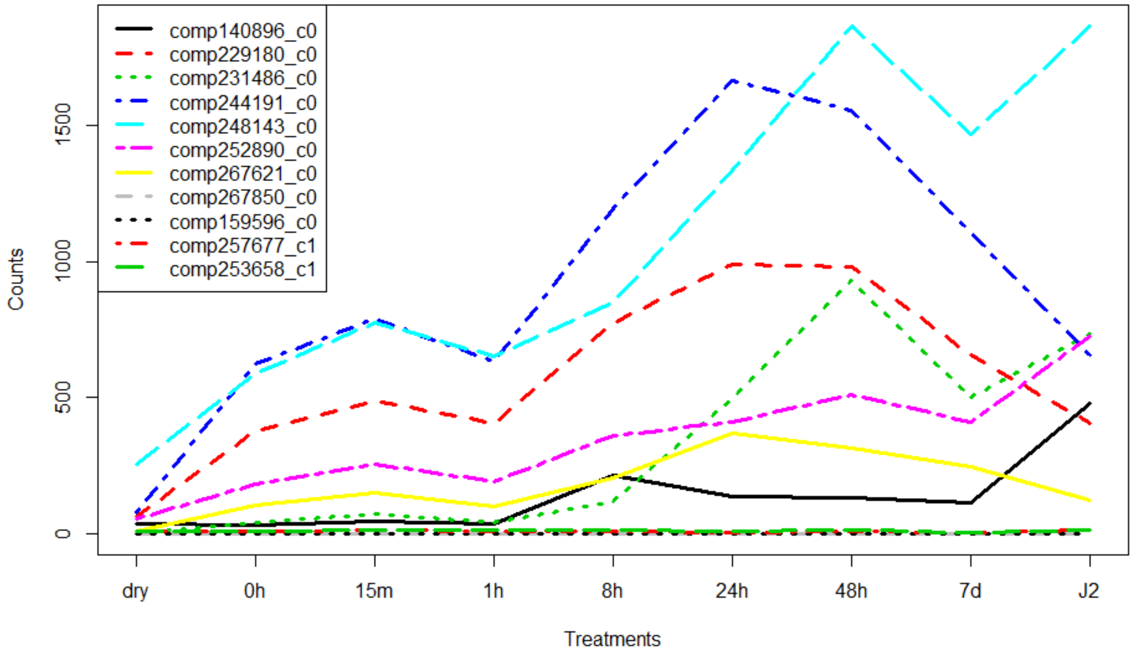

Figure S1: Expression pattern of 11 neprilysin genes in *G. rostochiensis*. Trinity transcriptome was used to measure gene expression. Treatment are dry cysts (dry), hydrated eggs (0h), and hydrated eggs exposed to PRD for 15 min, 1h, 8h, 24h, 48h and 7 days and hatched J2.

Table S1. *Globodera rostochiensis* and *G. pallida* Trinity transcriptome assembly statistics.

Metric	*Globodera rostochiensis*	*Globodera pallida*
Paired-end reads (100 bp)	511,208,631	213,156,265
Normalized pairs (30 X)	41,045,675	7,903,581
Trinity transcripts	1,075,007	300,796
Trinity components	239,134	31,346
Final transcriptome	**93,089**	**31,346**
Longest contig (bp)	18,280	15,960
N50 (bp)	1,293	2,527
GC (%)	47	47

Table S2 to S5 See excel file:

S2 DEGs_Grost_Trinity_fold_blast(S2).xlsx

S3 DEGs_Grost_augustus_fold_blast(S3).xlsx

S4 DEGs_Gpal_Trinity_fold_blast(S4).xlsx

S5 DEGs_Gpal_augustus_fold_blast(S5).xlsx

Table S6: Cluster of transcripts from *G. rostochiensis* Trinity transcriptome with expression similar to *trehalose 6-phosphate synthase.*

Contig	up-regulated Treatment	BLAST	Fold Change hydrated cyst vs dry cyst
comp24112_c0	Dry	histone h3	1.9
comp224595_c0	Dry	mgc83793 protein	1.6
comp246692_c0	Dry	ring finger and ccch-type zinc finger domain-containing protein 2	4.0
comp251106_c0	Dry	tubulin polyglutamylase ttll11-like	2.0
comp254088_c1	Dry	serine palmitoyltransferase 2	1.7
comp256950_c0	Dry	ribosomal protein s7p s5e containing protein	1.3
comp266765_c0	Dry	NA	3.7
comp188635_c0	Dry	histone	1.9
comp226839_c0	Dry	protein cogc-8	1.6
comp248755_c0	Dry	NA	2.0
comp251314_c3	Dry	adenylate kinase 2	2.5
comp254759_c0	Dry	probable trans-2-enoyl-mitochondrial-like	2.0
comp257619_c0	Dry	transcription initiation factor iib	1.6
comp208184_c0	Dry	hypothetical protein Bm1_35525	4.3
comp236180_c0	Dry	cre-rle-1 protein	3.7
comp249325_c0	Dry	g10 protein	1.9

comp252340_c0	Dry	cytoplasmic intermediate filament protein	2.1
comp254763_c0	Dry	NA	1.9
comp257819_c0	Dry	probable dolichyl pyrophosphate glc1man9 c2 alpha-glucosyltransferase	2.8
comp220998_c0	Dry	cop9 signalosome complex subunit	1.9
comp238646_c0	Dry	NA	4.0
comp250328_c1	Dry	NA	1.9
comp253394_c0	Dry	g10 protein	1.7
comp256639_c0	Dry	NA	2.1
comp258202_c0	Dry	islet cell autoantigen 1	2.3
comp222505_c0	Dry	cytoplasmic intermediate filament protein	2.7
comp246505_c0	Dry	NA	1.6
comp250328_c0	Dry	NA	3.5
comp253978_c0	Dry	polypyrimidine tract binding protein	1.8
comp256753_c0	Dry	trehalose 6-phosphate synthase	3.2
comp258460_c2	Dry	NA	2.2

Table S7: Cluster of transcripts from *G. rostochiensis* Trinity transcriptome with putative expression patterns of genes involved in cyst survival.

**Contig**	**up-regulated Treatment**	**BLAST**	**Fold change dry cyst *vs* hydrated cyst**
comp89407_c0	Dry	NA	3.7
comp140507_c0	Dry	NA	3.0
comp204557_c0	Dry	Selenoprotein	6.1
comp232167_c0	Dry	hypothetical protein CRE_16869	2.0
comp248011_c0	Dry	NA	2.0
comp250515_c1	Dry	Annexin	2.3
comp255917_c0	Dry	lysine histidine transporter	2.1
comp256424_c1	Dry	Zinc finger protein	1.7
comp258276_c1	Dry	briggsae cbr-pap-1 protein	2.8
comp258560_c0	Dry	NA	2.8

Table S8: Cluster of transcripts from *G. rostochiensis* Trinity transcriptome with expression similar to *nep-1*.

Contig	Up-regulated Treatment	BLAST	Mean fold-change in up-regulated hatching treatments
comp91041_c0	48h-7d-J2	gland protein g20e03	4.9
comp140896_c0	8h-J2	protein nep-1	6.5
comp213450_c1	8h-J2	nucleoside diphosphate kinase	24.3
comp220622_c0	J2	Patched family protein	NA
comp226361_c0	J2	NA	NA
comp235689_c0	J2	heat shock protein hsp20	NA
comp241314_c0	J2	beta-endoglucanase-1 precursor	NA
comp242450_c0	7d-J2	major facilitator superfamily domain-containing protein 8	NA
comp249021_c0	48h-J2	NA	5.3
comp251094_c0	J2	patched family protein	NA
comp263359_c0	J2	NA	NA

Table S9: Cluster of transcripts from *G. rostochiensis* Trinity transcriptome with putative expression pattern of genes involved in hatching.

**Contig**	**Up-regulated treatment**	**BLAST**	**mean fold change between up-regulated hatching treatment**
comp152061_c0	24h-48h	pectate lyase 2	8.3
comp178494_c0	48h	galactosylgalactosylxylosylprotein 3-beta-glucuronosyltransferase 2	12.1
comp252092_c1	48h	NA	9.2
comp79822_c0	24h-48h	pectate lyase 2	9.8
comp186241_c0	24h-48h	Peptidase M14, carboxypeptidase A domain and Proteinase inhibitor	6.1
comp220435_c0	24h-48h	Peptidase M14, carboxypeptidase A domain and Proteinase inhibitor	4.9
comp143987_c0	24h-48h	fatty acid elongation protein 3	7.0
comp205597_c0	24h-48h	carbonate dehydratase	5.3
comp179924_c0	24h-48h	ABC transporter substrate-binding protein	6.5
comp204845_c0	48h	lysosomal acid phosphatase	5.3
comp146670_c0	24h-48h	pectate lyase 1	5.9
comp236889_c0	24h-48h	general substrate transporter and Major facilitator superfamily domain-containing protein	6.1
comp197008_c0	24h-48h	ABC transporter substrate-binding protein	6.7

Table S10: Description of primers used in this study.

Gene symbol	Gene description	Forward primer	Reverse primer	Amplicon length (bp)	Amplification efficiency
Pmp-3	Putative membrane transporter	CTGGTTGCTGAGCAGGATAA	GATGAAGCCCGATTGGTAGAA	102	1.92
AaRS	Aminoacyl tRNA synthetase	CGGATTTACGGACCTTGTCTAC	GGGAATCCGTCACGCTTAAT	84	1.98
GR	Glutathione reductase	TTGAGAGACCATGCCGATTAC	GAGTTGAGACGCCGAATGT	102	1.90
Nep-1	Neprilysin	GCTGAAATGGTGGAGAAAGTG	TTTGACGCCCGAGTAGAAG	457	1.91
